# Successful treatment of critically ill chronic kidney disease patient with multi-organ dysfunction associated with H1N1 infection

**DOI:** 10.4103/0971-4065.78082

**Published:** 2011

**Authors:** V. B. Kute, P. R. Shah, K. R. Goplani, A. V. Vanikar, H. L. Trivedi

**Affiliations:** Department of Nephrology and Clinical Transplantation, Transfusion Services and Immunohematology, (ITS)- (IKDRC), Ahmedabad, Gujarat, India; 1 Department of Pathology, Laboratory Medicine, Transfusion Services and Immunohematology, (ITS)- (IKDRC), Ahmedabad, Gujarat, India

**Keywords:** CKD, H1N1, multi-organ dysfunction, oseltamivir

## Abstract

Chronic kidney disease (CKD) patients are at higher risk of H1N1 influenza A infection and associated complications. To our knowledge, this is the first case report of a febrile CKD patient with multi-organ dysfunction and associated H1N1 virus infection successfully treated with oseltamivir, hemodialysis, and mechanical ventilation. Oseltamivir is safe, effective, and well tolerated in our CKD patient.

## Introduction

Originating in Mexico in April 2009, influenza A/H1N1 2009 virus has spread worldwide.[1] As of February 28, 2010, more than 213 countries have reported laboratory-confirmed cases of H1N1 infections including at least 16 455 deaths.[2] World Health Organization (WHO) declared it a pandemic in June, 2009.[2] Samples from 130 008 persons have been tested for H1N1 in government and few private laboratories across the country in India, 29 880 (22.9%) of them have been found positive and 4.6% lab-confirmed cases have died.[1] There is limited information for chronic kidney disease (CKD) population, who is at a high-risk of H1N1-associated complications. The diagnosis of influenza is likely to be missed until CKD patient failed to respond to fluid removal and antibiotics. To our knowledge, we report the first case of severe H1N1 infection with multi-organ dysfunction in CKD successfully treated with antiviral therapy (oseltamivir), hemodialysis (HD), and mechanical ventilation.

## Case Report

A 67-year-old man was admitted with fever, cough, sore throat, breathlessness, muscle and joint pains of 1-week duration. He was a case of CKD stage 4 with hypertension (treated with amlodipine 5 mg twice a day) and diabetes mellitus of 1-year duration (on regular insulin, 5 IU three times a day).

On examination, he was obese (body mass index - 31 kg/m^2^) with blood pressure of 112/57 mmHg, temperature 39°C, respiratory rate of 36 breaths per minute, heart rate of 116 beats per minute, and oxygen saturation of 72% on room air which improved on high flow oxygen mask to 94%. The chest radiograph (posthemodialysis) revealed bilateral upper and middle zone pulmonary infiltrates.

Laboratory investigations revealed hemoglobin, 9.6 gm/L; total white cell count, 15.6 × 10^3^/*μ*l (differential count: 86% neutrophils, 6% lymphocytes, 6% monocytes, and 2% eosinophils); platelet count, 1.2 × 10^5^/*μ*l; serum creatinine (SCr), 7.8 mg/dl; alanine aminotransferase, 84 units/l (normal range: 0 – 40 units/l); aspartate aminotransferase, 176 units/l (normal range: 5 – 34 units/l); serum bilirubin, 3.5 mg/dl; serum albumin, 2.5 gm/dl; creatine phosphokinase, 900 U/l (normal range: 15 – 105 U/l); lactate dehydrogenase, 302 IU/l (normal range: 100 – 190 IU/l); lactate, 5.2 mmol/l (normal range: 0.4-2.0 mmol/l); and fasting/postprandial blood sugar of 130/180 mg/dl. Multiple blood, urine, and sputum cultures were sterile. On routine checkup 1 month prior to admission, his creatinine was 3 mg/dl. He was treated with antibiotics (Imipenem-cilastatin and clindamycin) and urgent HD during which 2 l of fluid was removed. An additional 2 l fluid was removed during a second HD. However his respiratory status did not improve, clinical condition deteriorated with rising body temperature, worsening hypoxia and dyspnea. He was transferred to the intensive care unit, intubated, and mechanically ventilated on day 2 of admission. Arterial partial pressure of oxygen/fraction of inspired oxygen was 125 mmHg. Acute Physiology and Chronic Health Evaluation (APACHE) II, Sequential Organ Failure Assessment (SOFA) scores, Multiple Organ Dysfunction Score, and Murray scores were 33, 12, 13, and 2.75, respectively. H1N1 virus was identified by reverse transcription polymerase chain reaction from his nasal and throat swabs. Oral oseltamivir, 75 mg on alternate days and amantadine 100 mg BID were initiated and continued for 10 days. He had history of exposure to H1N1 in community, and had not been vaccinated with seasonal influenza/pneumococcal vaccine within the last year. He received alternate day HD, and ionotropes were administered for 2 days. Repeat sputum culture on day 6 did not identify pathogenic organisms. On clinical improvement, he was extubated on day 6 and discharged on day 12. On follow-up three weeks later, his SCr was 3.3 mg/dl (CKD stage 4) and urine output was 1.2 l/day. He did not require dialysis after discharge. No adverse effects of oseltamivir were noted.

Protective measures like extensive use of surgical masks and N 95 respirators in addition to goggles, gowns, and gloves, strict application of universal precautions as well as liberal use of gel-alcohol hand sanitizer, limitation and control of entries; prompt isolation of suspected cases were implemented in order to limit the spread of the infection in dialysis units. All close contacts including health care workers received oseltamivir (75 mg for 10 days) and did not develop any influenza like illness.

## Discussion

The presence of diabetes, obesity, respiratory/heart/liver diseases, immunosuppression, and CKD has been shown to be associated with higher mortality in H1N1 pandemic.[3] CKD patients mainly present with fever, cough, muscle and joint pains like any healthy patient.[4,5] In our patient, the initial clinical presentation of infection did not differ from those who were nonimmunocompromised, and he was at high risk for H1N1 due to associated comorbid condition.

A thorough knowledge of dosing schedule of oseltamivir is mandatory to avoid undesirable side effects. In patients with severe pneumonia, the recommended dose is twice standard dosing (150 mg BID in adults), and longer treatment duration (10 days vs 5 days) is required due to the potential for decreased enteral absorption among critically ill patients and high and prolonged viral replication in lower respiratory tract.[6] A 30-mg dose of oseltamivir given once weekly in continuous ambulatory peritoneal dialysis or after alternate sessions in HD provides sufficient exposure to oseltamivir to allow safe and effective anti-influenza treatment and prophylaxis.[7] Because oseltamivir is eliminated by kidneys, a reduced dose of 75 mg orally three times weekly after HD is recommended with low-flux dialysers.[7] With high-flux dialysers, increased dose of 75 mg daily for 5 days is recommended.[5] Patients who have severe or progressive clinical illness due to oseltamivir-susceptible and M2 inhibitor-susceptible virus may be treated with both oseltamivir and amantadine/rimantadine.[6] We used oseltamivir, 75 mg on alternate days (high dose according to glomerular filtration rate (GFR) for 10 days in our patient along with amantadine. He had mild gastrointestinal complaints of intermittent nausea and vomiting that were manageable. Given a potentially mild adverse effect of treatment from our experience and that of others, a higher dose of oseltamivir is a potential option for complicated H1N1 infection in CKD.[8]

Peritoneal dialysis is an attractive option as dialysis can be done at home even during a pandemic.[9] We dialyzed our patient in isolated room. Mortality in H1N1 is associated with higher APACHE, SOFA, Murray scores, a higher oseltamivir dose, lower oxygen inspired fraction/alveolar pressure ratio, thrombocytopenia, hypoalbuminemia, and acute renal failure.[10] Mortality among the patients requiring mechanical ventilation is reported in 58% patients.[11] Our patient was successfully treated despite above risk factors and multi-organ dysfunction.

Our patient had CKD stage 4 with acute kidney injury (AKI) due to H1N1 infection and he recovered after successful treatment. AKI is multifactorial, due to hypoxaemia, hypoperfusion, renal vasoconstriction, and rhabdomyolysis in the setting of a severe systemic inflammatory response syndrome.[12,13] Although to date there is no documented direct cytopathic injury in kidneys of patients with influenza, some authors have suggested it as cause of AKI.[13]

In critically ill patients with pandemic H1N1, renal injury and the need for dialysis are common and associated with increased mortality and length of intensive care unit stay.[11,12] Centers for Disease Control and Prevention and WHO warn that pandemic H1N1 poses an extra risk for those with health problems like CKD and these patients should be considered for H1N1 vaccination.[4,14–19] Our report supports this recommendation. Uremia-induced immune dysfunction[20] might lead to the atypical clinical presentation in a dialysis patient. H1N1 influenza A is an important differential diagnosis in dialysis patients who are short of breath or febrile.[5]

To our knowledge, this is the first case report from India on successful treatment of critically ill CKD patient with multi-organ dysfunction and associated H1N1 influenza A virus infection. Oseltamivir is safe, effective, and well tolerated in our CKD patient.
